# Nanoporous Microneedle Arrays Effectively Induce Antibody Responses against Diphtheria and Tetanus Toxoid

**DOI:** 10.3389/fimmu.2017.01789

**Published:** 2017-12-13

**Authors:** Anne Marit de Groot, Anouk C. M. Platteel, Nico Kuijt, Peter J. S. van Kooten, Pieter Jan Vos, Alice J. A. M. Sijts, Koen van der Maaden

**Affiliations:** ^1^Department of Infectious Diseases and Immunology, Faculty of Veterinary Sciences, Utrecht University, Utrecht, Netherlands; ^2^MyLife Technologies, Leiden, Netherlands

**Keywords:** nanoporous microneedles, intradermal vaccination, antigen release, humoral immune response, diphtheria, tetanus

## Abstract

The skin is immunologically very potent because of the high number of antigen-presenting cells in the dermis and epidermis, and is therefore considered to be very suitable for vaccination. However, the skin’s physical barrier, the stratum corneum, prevents foreign substances, including vaccines, from entering the skin. Microneedles, which are needle-like structures with dimensions in the micrometer range, form a relatively new approach to circumvent the stratum corneum, allowing for minimally invasive and pain-free vaccination. In this study, we tested ceramic nanoporous microneedle arrays (npMNAs), representing a novel microneedle-based drug delivery technology, for their ability to deliver the subunit vaccines diphtheria toxoid (DT) and tetanus toxoid (TT) intradermally. First, the piercing ability of the ceramic (alumina) npMNAs, which contained over 100 microneedles per array, a length of 475 µm, and an average pore size of 80 nm, was evaluated in mouse skin. Then, the hydrodynamic diameters of DT and TT and the loading of DT, TT, and imiquimod into, and subsequent release from the npMNAs were assessed *in vitro*. It was shown that DT and TT were successfully loaded into the tips of the ceramic nanoporous microneedles, and by using near-infrared fluorescently labeled antigens, we found that DT and TT were released following piercing of the antigen-loaded npMNAs into *ex vivo* murine skin. Finally, the application of DT- and TT-loaded npMNAs onto mouse skin *in vivo* led to the induction of antigen-specific antibodies, with titers similar to those obtained upon subcutaneous immunization with a similar dose. In conclusion, we show for the first time, the potential of npMNAs for intradermal (ID) immunization with subunit vaccines, which opens possibilities for future ID vaccination designs.

## Introduction

The skin has great potential for vaccine delivery, because it is a large organ that is easy to reach. Delivery *via* the skin circumvents degradation challenges to biomacromolecules, as posed, for example, by the gastrointestinal delivery route (1, 2). The skin, with the stratum corneum as outer barrier, is designed to keep foreign materials including pathogens out of the body. Besides, the skin is immunologically very potent, with various professional antigen-presenting cells, such as dermal dendritic cells and Langerhans cells (3, 4), present in the dermis and epidermis, respectively. To circumvent the barrier function of the stratum corneum and reach antigen-presenting cells for vaccination purposes, microneedles can be used. Microneedles are needle-like structures with a length in the micrometer range and are a promising tool to deliver drugs and vaccines across the barrier. Furthermore, they represent a possible painless vaccination method (5), they present reduced contamination risks compared with traditional needles, they allow for injection by less trained personnel and even have potential for self-administration (6). However, microneedles need to be sufficiently long and strong enough to pierce the stratum corneum, but also preferably short enough to not reach the nociceptors. Various microneedles are under development, which are hollow-, solid-, dissolving-, or less known porous structured (6–10). For all types, multiple strategies have been investigated for the delivery of vaccine antigens into the skin, as reviewed by van der Maaden et al. (10).

Porous microneedles, which may be used as a single-unit-drug delivery system, can be prepared from pore-forming materials (11), from (nano)particles (12, 13), or by making solid microneedle material porous (14, 15). Porous microneedle arrays (MNAs) can be loaded with a drug, by loading the formulation into the pores of the MNAs. The drug is released when the microneedles are pierced into the skin *via* diffusion from the pores. To date, several materials have been used for the production of porous MNAs. Among these are biodegradable polymeric porous MNAs with a porosity of 75%, which, however, lack the strength to penetrate the skin (11). When using a brittle material, like silicon, the pores that are introduced in the material need to be sufficiently small to provide enough strength for skin piercing (14, 15). The use of porous silicon material, therefore, is limited to the delivery of low-molecular weight drugs (10). Using self-setting ceramics for production of porous MNAs increases MNA strength. However, drug loading into these MNAs requires circumstances that are unfavorable for formulating biomacromolecules, because it involves exothermic reactions or organic solvents (ethanol) (16).

In this study, microneedles composed of a biocompatible ceramic, alumina (Al_2_O_3_) (12), were tested for their suitability for intradermal (ID) vaccination. With an average pore size of 80 nm and an estimated porosity of 40%, these microneedles allow for encapsulation of large biomacromolecules before production (10, 13). In previous studies, it was shown that alumina nanoporous microneedle arrays (npMNA) can be successfully loaded with small molecules or nanoparticles with sizes up to 100 nm in solution or dispersion *via* absorption (*via* capillary forces), respectively, and to release these substances *in vitro* by diffusion. The npMNAs had sufficient strength to reproducibly pierce *ex vivo* human skin without breaking (10) and have shown to activate immune cells upon dermal application of peptide-loaded npMNAs in a murine model (17). However, characterization of ceramic alumina npMNAs loaded with larger, more relevant molecules, such as subunit vaccine antigens, had not been performed so far.

In this study, characterization and application of alumina npMNAs are described. Loading of npMNAs with diphtheria toxoid (DT) and tetanus toxoid (TT), antigen release in murine skin *ex vivo*, and *in vivo* immunogenicity of npMNA-delivered antigens were examined. We show that npMNA-mediated vaccine delivery elicits TT- and DT-specific antibody responses in mice, comparable to those induced by subcutaneous (SC) immunization with a similar dose. This is the first report showing the potential of porous microneedles in dermal immunization with subunit vaccines.

## Materials and Methods

### Materials

Diphtheria toxoid and TT (for *in vitro* assays) were obtained from Staten Serum Institute (Copenhagen, Denmark) and imiquimod (IMQ) Vaccigrade was obtained from Invivogen. Trifluoroacetic acid (TFA), 3,3′,5,5′-tetramethylbenzidine (TMB) and bovine serum albumin (BSA), and 0.4% (w/v) were obtained from Sigma Aldrich. High-performance liquid chromatography (HPLC)-R grade acetonitrile (ACN) was from Biosolve and phosphate-buffered saline (PBS; pH 7.2, 1.5 mM KH_2_PO_4_, 2.7 mM Na_2_HPO_4_–7H_2_O, and 155 mM NaCl) was from Gibco (ThermoFisher Scientific). IRdye800cw carboxylic acid *N*-hydroxy succinimide ester (IRdye800cw-NHS) was purchased from Li-cor (Lincoln, NE, USA). Dexdomitor was purchased from Orion Corporation, Narketan ketamine from Vétoquinol, and Atipam was purchased from Dechra. Goat anti-mouse immune globulin G (IgG) total horseradish peroxidase (HRP) (GaM-IgG total HRP), goat anti-mouse IgG1 HRP (GaM-IgG1 HRP), and goat anti-mouse IgG2a HRP (GaM-IgG2a HRP) were obtained from Southern Biotech and microtiter plates 9610 used for ELISA were from Corning Costar.

### Preparation and Characterization of npMNA

**Figure 1:**
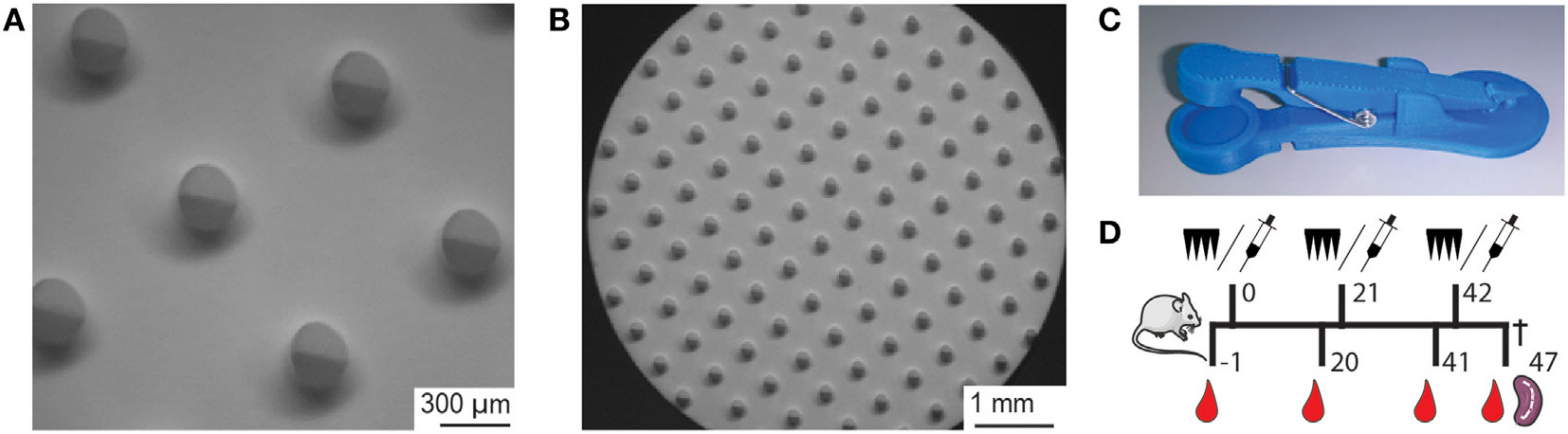
**(A,B)** Brightfield microscopy images of a nanoporous microneedle array (npMNA) from the needle-side with microneedles with a length of 475 µm and a density of 105 microneedles/array. **(C)** Microneedle applicator design that was used to apply npMNAs onto mouse ears. Upon lowering the applicator lid, a microneedle array is pierced into the skin by impact application, and the npMNA is subsequently held onto the skin by force (4 N). **(D)** Experimental setup for immunization studies. Immunization with either npMNA or hydrodermic needle on days 0, 21, and 42. Blood samples for serum were collected on days −1, 20, 41, and 47, at which the experiment was terminated and subsequently, spleens were collected.

Nanoporous microneedle arrays were produced by using a double replication technology as previously described (12). In brief, from an inverse silicon master a first positive PDMS mold was created, from which a second inverse PDMS mold was produced. Alumina npMNAs were fabricated at LouwersHanique B.V. from the second PDMS mold according to MyLife Technologies’ proprietary manufacturing procedure (18), using a slurry that contains alumina nanoparticles with an average size of 300 nm and a plasticizer. After controlled drying, the resulting MNAs were removed from the PDMS mold and were sintered at 1,450°C. This results in removal of the plasticizer and the formation of nanoporous alumina material with an average pore size of 80 nm and a porosity of approximately 40% (10, 12). Microneedles used in this study had a length of 475 µm and a density of 150 microneedles/cm^2^ on a back plate of 0.7 cm^2^ (105 microneedles/array; Figures 1A,B). The total volume in the nanopores of only the tips of the microneedles of a single MNA was calculated to be 0.25 µL. Bruker Nano Surface analysis was performed to characterize the geometry of the npMNAs.

### Preparation of npMNAs with Antigen-Loaded Microneedle Tips

To only load the tips of the microneedles, microneedles were pierced through a foil (Parafilm^®^) by using a UAFM-V1 electrical applicator (uPRAX Microsolutions) at a velocity of 65 cm/s. Next, a drop of 5 µL drug formulation was applied onto the foil-pierced npMNAs to absorb a drug/vaccine formulation into the microneedle tips. After 5 s, the surplus drop of drug formulation and the foil were sequentially removed from the npMNAs. To confirm that only the microneedle tips can be loaded with a drug formulation, the tips of npMNAs were loaded with a 0.4% trypan blue solution as described earlier.

### Skin Penetration

To test the piercing ability, a npMNA was applied trice on the dorsal side of *ex vivo* murine ears (Balb/C), which were collected from surplus mice, by using 3D-printed uPRAX impact applicators (Figure 1C) having an average holding force of 4.08 ± 0.75 N (mean ± SD, *n* = 19). Subsequently, the three pierced *ex vivo* mouse ears were incubated with 50 µL of a 0.4% trypan blue solution at room temperature. After 30 min, the trypan blue was removed, and the ears were washed in 10 mL PBS. Finally, the blue dots (piercings) were counted, from which the penetration efficiency was calculated.

### Hydrodynamic Diameter and Size Distribution of Antigens

To determine whether the npMNAs are suitable devices to load and release subunit antigens DT and TT, the hydrodynamic diameter and size distribution of DT and TT were determined by using dynamic light scattering on a Zetasizer Nano (Malvern Instruments). For these measurements, DT and TT were at a concentration of 0.8 and 0.4 mg/mL, respectively.

### Release of IMQ and Antigen from Nanoporous Alumina *In Vitro*

Imiquimod has the potential to enhance the immunogenicity of antigens in the skin (19). To evaluate how IMQ is released from IMQ-loaded nanoporous material in the presence of DT and TT, npMNAs were loaded by applying a drop (on the microneedle side of a npMNA that were not pierced through a foil) of 5 µL PBS containing only 2.5 µg IMQ, or 2.5 µg IMQ and 2.5 Lf DT or TT. Such a drop is absorbed into an npMNA within seconds because of capillary forces. Next, the IMQ-loaded npMNAs were incubated in 2.5 mL release buffer (PBS) under shaking at 300 rpm, and samples of 75 µL were taken in duplicate at different time points (1, 5, 10, 30, 60, 120, and 240 min). The IMQ concentration in the release buffer was determined by using HPLC analysis on an Agilent 1100 series HPLC equipped with a UV detector using a Phenomenex Kinetex 150 mm × 4.6 mm 2.6 μm EVO C18 column. A linear gradient of 5% solvent A (ACN with 0.1% TFA) to 68% solvent B (milliQ with 0.1% TFA) from 0 to 12 min was detected at a wavelength of 242 nm at a retention time of 9.8 min.

To assess the release of antigen from antigen-loaded nanoporous material, npMNAs (that were not pierced through a foil) were loaded with 15 µL PBS that contained 5 Lf DT or TT as described earlier. Next, antigen-loaded npMNAs were incubated in 4 mL release buffer while shaking at 300 rpm. At different time points (1, 5, 10, 30, 60, 120, and 240 min), samples of 500 µL were taken in which the released amount of antigen was quantified by measuring the intrinsic fluorescence (emission wavelength of 348 nm) at an excitation wavelength of 280 nm and using standard concentrations of DT and TT ranging from 0.01 to 50 ng/mL on a Tecan Infinite M1000 plate reader. The release of antigen in the presence of IMQ was not investigated, because IMQ is fluorescent at similar wavelengths (excitation at 260 nm and emission at 340 nm) that are used to measure the intrinsic fluorescence of the antigens (20).

### Fluorescently Labeling of Antigens

To quantify the amount of DT and TT that is delivered from DT- and TT-loaded npMNAs into skin, DT and TT were labeled with a near-infrared fluorescent dye (IRdye800cw-NHS). To this end, 1 mg/mL solutions of DT and TT in a 100 mM carbonate buffer pH 8.5 were prepared. Subsequently, 1 mL of each of these solutions was added to 500 µg of IRdye800cw-NHS. After 1 h shaking (300 rpm) at 37°C, the free dye was removed and the carbonate buffer was exchanged by PBS using a Zeba™ spin desalting column with a molecular weight cutoff (MWCO) of 7 kDa (Thermo Fisher Scientific). Next, IRdye800cw-labeled antigens were concentrated approximately 50 times by using 0.5 mL Amicon (Millipore) 10 kDa MWCO filters. Finally, the concentration of IRdye800cw-labeled DT and TT was determined by using a calibration curve of non-labeled antigens and measuring the intrinsic fluorescence (as described earlier).

### Quantification of Antigen in *Ex Vivo* Mouse Ears

To quantify the delivered amount of DT and TT into murine skin, npMNAs of which only the tips were loaded (using foil piercing) with fluorescently labeled antigens were prepared by using 5 µL of 12 Lf/μL (DT) and 6 Lf/μL (TT), as described earlier. The microneedles were applied by impact application and retained onto the skin by using a uPRAX 3D-printed applicator. After 30 min at room temperature, the antigen-loaded npMNAs were removed from the ears, and their fluorescence was compared with the fluorescence of standard solutions having known amounts of fluorescently labeled DT and TT, by using a IVIS^®^ lumina II equipped with an ICG filter set. The intradermally delivered amounts of DT and TT were quantified by using Living Image^®^ software (version 4.3.1).

### Preparation of Vaccine Formulations for Loading npMNAs for Immunization

Subunit vaccine formulations of DT and TT for loading the microneedle tips were prepared from antigen stock solutions (2.0 and 0.7 Lf/μL, respectively). Antigen stock solutions were concentrated (6–30×) by using 0.5 mL Amicon (Millipore) 10 kDa MWCO filters. Next, the concentration of the concentrates was determined by measuring the intrinsic fluorescence as described earlier. Finally, the antigen concentration was adjusted by diluting the concentrates in PBS to a concentration of 12 and 6 Lf/μL for DT and TT, respectively.

### Immunization of Mice

Seven-week-old Balb/C female mice (10 mice per group) obtained from Charles River (France) were immunized with 1.2 Lf (~0.50 μg) DT and 1.5 Lf (~0.77 μg) TT, or with 0.6 Lf DT and 0.75 Lf TT adjuvanted with 0.5 µg IMQ, on days 0, 21, and 42. The vaccine was administered *via* a SC injection of 100 µL in the neck using traditional hypodermic needles, or by dermal administration in the ear pinnae by using microneedles of which only the tips were loaded with vaccine formulation. Before each microneedle-based immunization, mice were anesthetized with 30 mg/kg ketamine and 0.1 mg/kg Dexdomitor by intraperitoneal injection. After the microneedles were removed, the anesthetic was antagonized with 0.4 mg/kg Atipam. On each ear, a DT- and TT-loaded npMNA was applied for 30 min by using a uPRAX applicator (Figure 1C). As a negative control mice were mock immunized *via* a PBS-loaded npMNA. Blood samples were collected from the tail vein 1 day before each immunization and serum was obtained by centrifugation; spleens were collected at day 47 (Figure 1D).

### DT- and TT-Specific IgG Total, IgG1, and IgG2a Titers

Diphtheria toxoid- and TT-specific antibody titers were determined using ELISA. ELISA plates were coated with 0.2 µg DT or 0.2 µg TT for 30 min and then blocked with 1% BSA in PBS for 15 min. Thereafter, 50 µL of serum sample at dilutions ranging from 1:25 until 1:200 (day 0), or from 1:200 until 1:25,000 (day 20, 41, and 47) were added, for 30 min. After extensive washing with 0.01% Tween 20 in PBS (PBST), wells were incubated for 30 min with GaM-IgG total HRP (1:5,000), GaM-IgG1 HRP (1:3,000), or GaM-IgG2a HRP (1:5,000). After extensive washing with PBST, antibody titers were quantified by adding 50 µL of stock TMB. Reactions were stopped after 60 s, with 100 µL of 1 M H_2_SO_4_, and the absorption was measured at a wavelength of 450 nm, with a reference wavelength of 650 nm, on a Microplate reader 550 (Bio-Rad). Titers of all animals at all time points for each isotype were measured in one experiment per antigen.

### Statistical Analysis

From 4 optical density (OD) values (at a wavelength of 450 nm) of diluted serum samples, the EC_50_ midpoint titers were determined using GraphPad Prism (GraphPad Software Inc., San Diego, CA, USA, v6.05). Immunized mice showing OD values below the mean OD values + three times the SD measured for PBS treated mice were considered as non-responders and were given an arbitrary value of 0, equal to ^10^Log value of 1. Statistical differences between immunization groups were determined using a non-parametric one-way ANOVA Kruskal–Wallis test with a Dunn’s test for multiple comparisons, and statistical significance was presented as follows: *p < 0.05; **p < 0.01; ***p < 0.001, and ns, not significant. Ratio of IgG1:IgG2a was determined by dividing midpoint titers of individual isotypes, and if animals were considered as non-responders for one of the isotypes (see above), they were excluded from ratio analysis.

## Results

### Characteristics of npMNA

**Figure 2:**
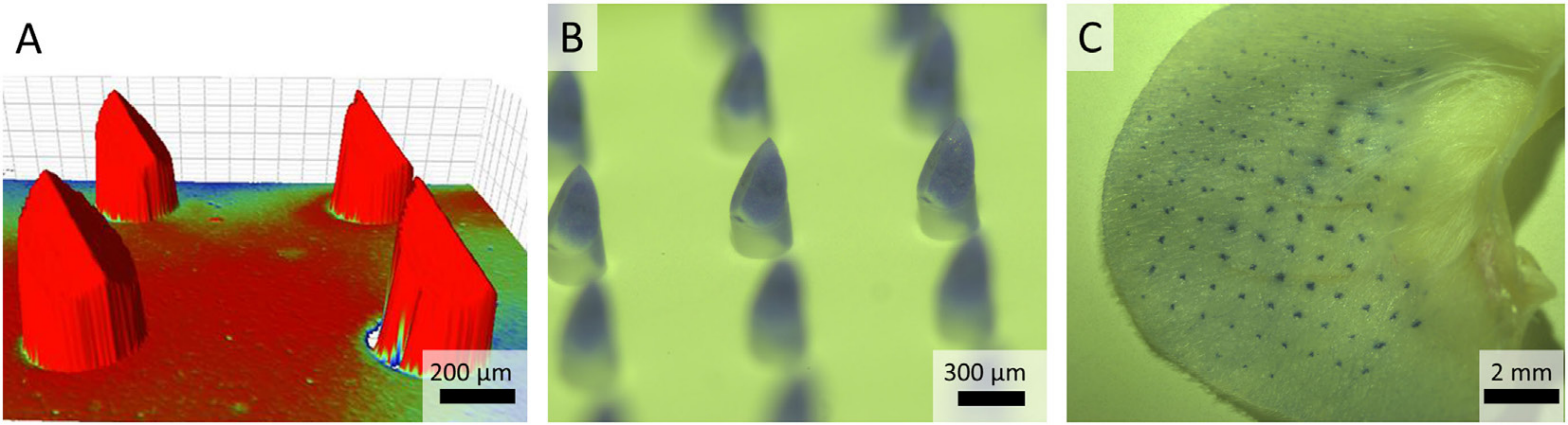
**(A)** Bruker analysis was used for geometry and surface analysis and to measure the distance between the microneedle backplate and microneedle tip. The color is indicative for the size of the substrate-fillable microneedles. **(B)** Brightfield microscopy images of a nanoporous microneedle array (npMNA) of which only the microneedle tips are loaded with a trypan blue solution. **(C)** Representative image of a trypan blue piercing assay of *ex vivo* murine ears with an npMNA using the uPRAX applicator.

Nanoporous microneedle arrays fabricated from alumina nanoparticles as previously reported (10), were characterized for geometry and dimensions *via* surface Bruker analysis, which showed that the ceramic microneedles had an average length of 475 µm and a needle shaft diameter of 275 µm (Figure 2A). For economic reasons, loading of only the tip of the microneedle with vaccine is an advantage, as residual vaccine quantities in the npMNAs will be strongly reduced. To investigate the possibility to only load the microneedle tips, npMNAs were pierced through foil and tips were subsequently loaded with a trypan blue solution for visualization. Brightfield microscopy showed successful tip loading and no loading of the backplate of the npMNA (Figure 2B).

### Strength of npMNA by Skin Penetration

**Figure 3:**
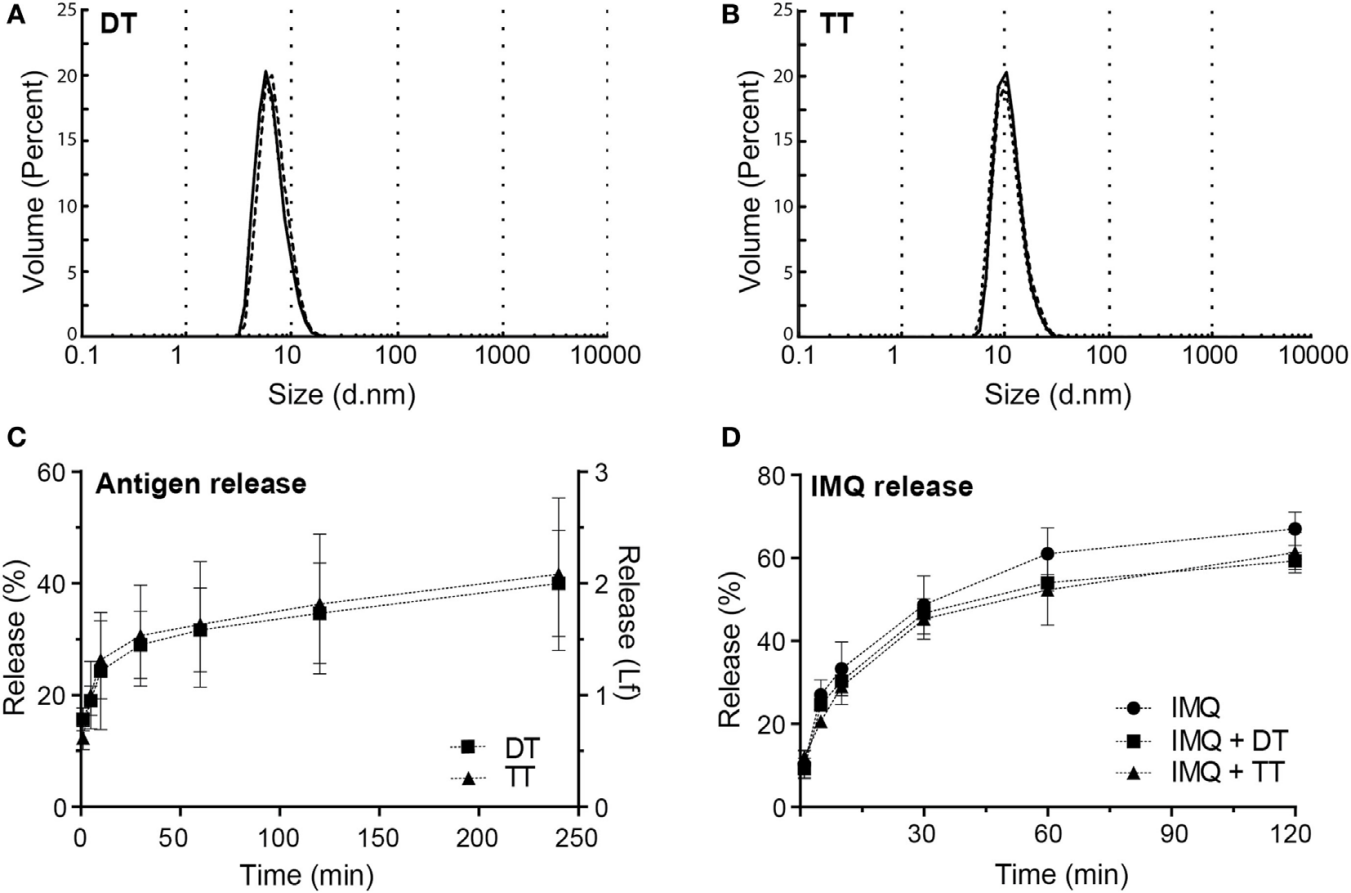
**(A)** Hydrodynamic diameter of diphtheria toxoid (DT) (8.72 ± 2.83 nm, mean ± SD, *n* = 3). **(B)** Hydrodynamic diameter of tetanus toxoid (TT) (13.5 ± 5.6 nm, mean ± SD, *n* = 3). **(C)** Release of DT and TT in release buffer measured by intrinsic fluorescence **(D)** release of imiquimod (IMQ) from nanoporous microneedle arrays in phosphate-buffered saline measured by high-performance liquid chromatography.

The ability of npMNAs to penetrate the skin is essential for ID antigen delivery. To determine whether the npMNAs are strong enough to penetrate the skin effectively and reproducibly, skin piercing was evaluated in *ex vivo* murine ear skin using a trypan blue assay (Figure 2C). Using the npMNAs resulted in an average piercing efficiency of 87 ± 17% (mean ± SD, *n* = 3). No visual breakage or reduction in microneedle strength or sharpness was observed. Together, these data show that the developed npMNA can be used to repeatedly penetrate the skin without breakage. To determine whether the npMNAs, having an average pore size of 80 nm, are suitable to be loaded with subunit vaccine antigens, the hydrodynamic diameter of DT and TT were determined by using dynamic light scatter. This revealed that DT and TT had a hydrodynamic diameter of 8.7 ± 2.8 nm (mean ± SD, *n* = 3) and 13.5 ± 5.6 nm (mean ± SD, *n* = 3), respectively, (Figures 3A,B). Therefore, the npMNAs should be suitable to be loaded with DT and TT into their nanopores.

### Antigen and Adjuvant Loading and Release *In Vitro*

After the npMNAs were loaded with either one of the antigens, the release of these antigens from the npMNAs in a release buffer was determined by measuring the intrinsic fluorescence of the antigens. After antigen-loaded npMNAs were incubated in release buffer for 30 min, 30% of both DT and TT were released from the npMNAs (Figure 3C). Besides, the release of IMQ from IMQ-loaded npMNAs was quantified after incubating them in a release buffer and using HPLC with UV detection. This revealed that approximately 50% of the npMNA-loaded IMQ was released after 30 min. Furthermore, it was observed that the co-delivery of IMQ and DT or TT did not result in a decreased release rate (Figure 3D). The release of IMQ reached a plateau at 60%, which indicates that IMQ partially adsorbs onto the npMNA. This was confirmed by incubating non-loaded npMNAs in an IMQ-containing buffer, having the same amount of IMQ as the IMQ-loaded npMNAs. The concentration of IMQ in the buffer decreased from 100 to 60% over time, showing that 0.2 µg IMQ was adsorbed onto the npMNA surface (data not shown). The effect of IMQ on the release of DT or TT could not be assessed due to interference with the fluorescence of IMQ (20). Together, these data showed that ceramic alumina npMNAs are suitable to be loaded with the subunit vaccine proteins DT and TT and the adjuvant IMQ, and that the antigens and adjuvant are released *in vitro*.

### Release of Antigen into *Ex Vivo* Skin

**Figure 4:**
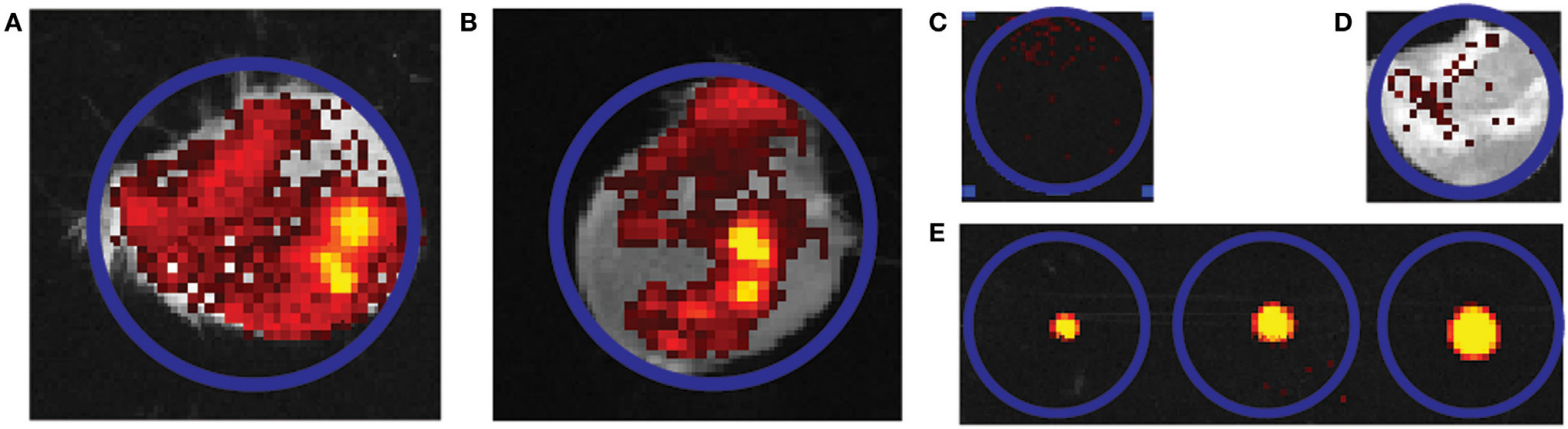
Representative quantification image of the delivery of fluorescently labeled antigen into mouse ears. **(A,B)** An overlay of picture of the mouse ear and infrared fluorescence imaging. Two independent ear piercing experiments are shown for diphtheria toxoid (DT) (*n* = 2) **(A)** and tetanus toxoid (*n* = 3) **(B)**. **(C,D)** Background fluorescence without and with mouse ear. **(E)** Gradient of solution containing 0.24, 0.6, and 1.2 Lf DT. Blue circles all indicate region of interest and have an equal diameter in all cases.

Next, the delivery of fluorescently labeled antigens from npMNAs into *ex vivo* murine skin was investigated. The antigen dose delivered into the skin was quantified after the application of the fluorescently labeled antigen-loaded npMNAs onto mouse ears. The delivery of the antigens into the ears was quantified by using infrared fluorescence imaging (Figures 4A,B) and was compared with a gradient of known amounts of fluorescently labeled antigens (Figures 4C–E). The delivery of DT was 0.61 ± 0.44 Lf (which corresponds with ~0.25 ± 0.18 μg) per MNA (Figure 4A), and the delivery of TT was 0.77 ± 0.23 Lf (~0.38 ± 0.11 μg) per MNA (Figure 4B).

Delivery efficiencies of DT and TT from the npMNAs were calculated by using the geometric values of the npMNAs. With an estimated total tip pore volume of 0.25 µL, the loaded amount of DT was calculated as 0.25 µL × 12 Lf/μL = 3 Lf/MNA. With a release of 0.61 Lf out of 3 Lf loaded, 20% delivery efficiency was achieved for DT, after 30 min of application onto the skin. For TT, an amount of 0.25 µL × 6 Lf/μL = 1.5 Lf/MNA was loaded and with a release of 0.77 Lf, the delivery efficiency was 0.77 Lf/1.5 Lf = 51%, after 30 min. For immunization studies, two arrays per mouse were used per antigen and this resulted in a delivery of 1.25 Lf (~0.5 μg) DT and a delivery of 1.53 Lf (~0.77 μg) TT. These values of delivered doses correspond with 26 and 31%, respectively, of the currently used human vaccination dose (5 Lf).

### Immune Response after Dermal Immunization

**Figure 5:**
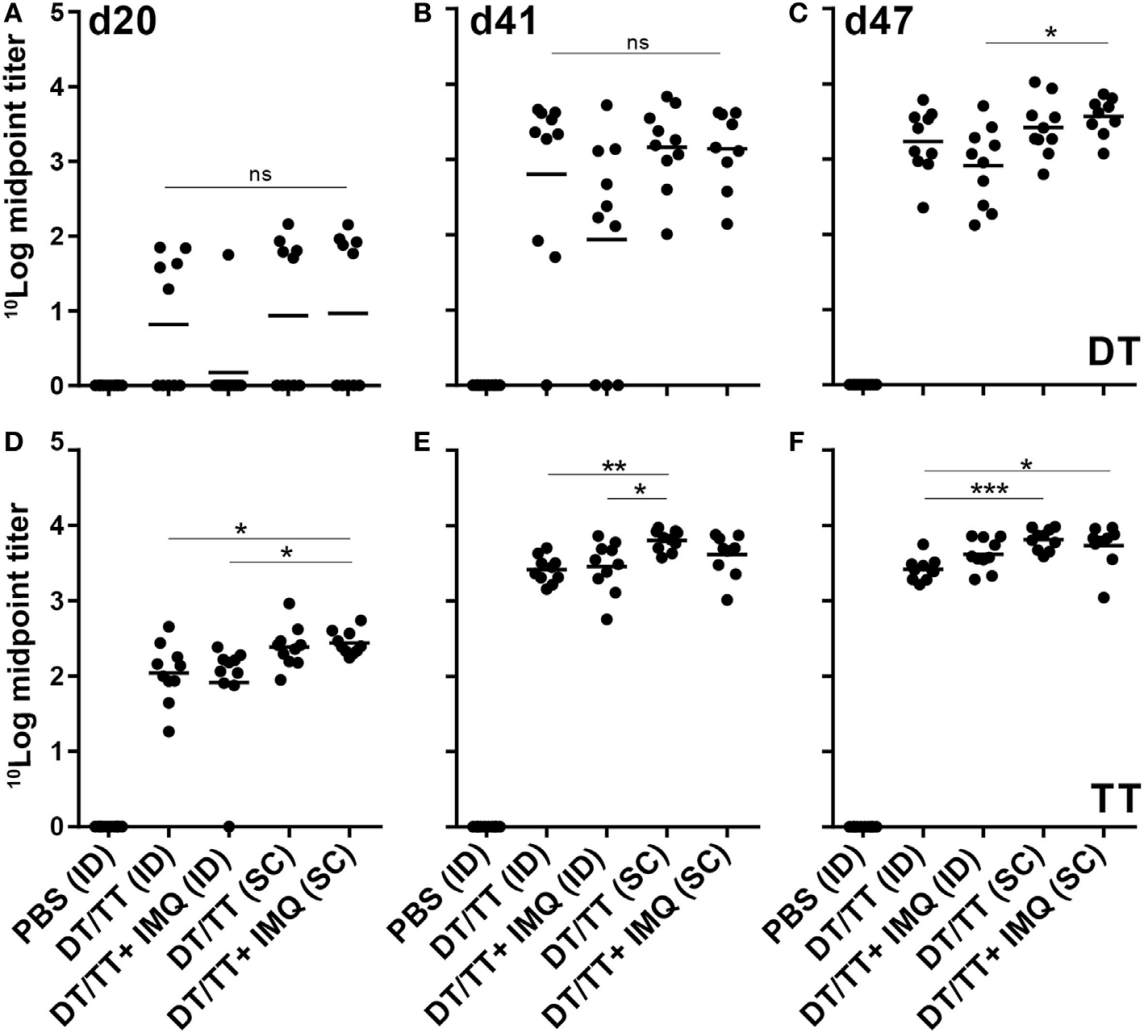
Serum immune globulin G (IgG) responses (mean + individual results) after immunization with phosphate-buffered saline or diphtheria toxoid (DT) and tetanus toxoid (TT) intradermal (ID) loaded onto nanoporous microneedle array or SC, both routes with or without imiquimod (IMQ). When IMQ was added, only half of the antigen dose was applied. IgG responses were detected against DT antigens **(A–C)** and TT antigens **(D–F)**. Kruskal–Wallis test with Dunn’s *post hoc* test was performed to determine statistical differences between midpoint titers determined using four different titers dilutions.

To determine whether npMNA-mediated ID delivery induces antigen-specific immunity, mice were immunized with both DT (1.2 Lf) and TT (1.5 Lf) using antigen-loaded npMNAs (ID administration) or using a needle and a syringe (SC injection). After each immunization, antibody titers in the serum were determined (Figure 1D). As expected, no DT- and TT-specific antibodies were detected 1 day before the first immunization (data not shown). At day 20 after immunization, approximately 50% of the immunized mice showed detectable IgG titers against DT, which were increased after the first boost measured at day 41. After the second boost (measured at day 47), all mice produced antibodies against DT (Figures 5A–C). IgG titers against TT were slightly higher compared with DT-specific titers (Figures 5D–F). When comparing ID administration with SC injection, no statistical differences were found for DT-specific titers. IgG titers against TT were slightly higher upon SC delivery as compared with microneedle-mediated delivery (significant after the boost and second boost) (Figures 5D–F).

For both immunization routes, also DT/TT combinations adjuvanted with toll-like receptor (TLR) 7 agonist IMQ were tested to determine whether adjuvants may modify the quality of vaccination-induced antibody responses. Antigen quantities loaded in combination with IMQ were half the antigen dose loaded into npMNAs in the absence of this TLR7 agonist (0.6 Lf DT, 0.75 Lf TT, and 0.5 µg IMQ). Despite the lower antigen dose, similar antibody titers were detected in mice immunized with adjuvanted compared with unadjuvanted vaccine, with the exception of DT-specific responses measured at day 41 where 3/10 in the adjuvanted group compared with 1/10 mice in the unadjuvanted group had failed to respond (Figure 5). Overall, observing TT-specific antibody titers, the subcutaneously injected mice showed slightly higher titers than the intradermally immunized mice.

**Figure 6:**
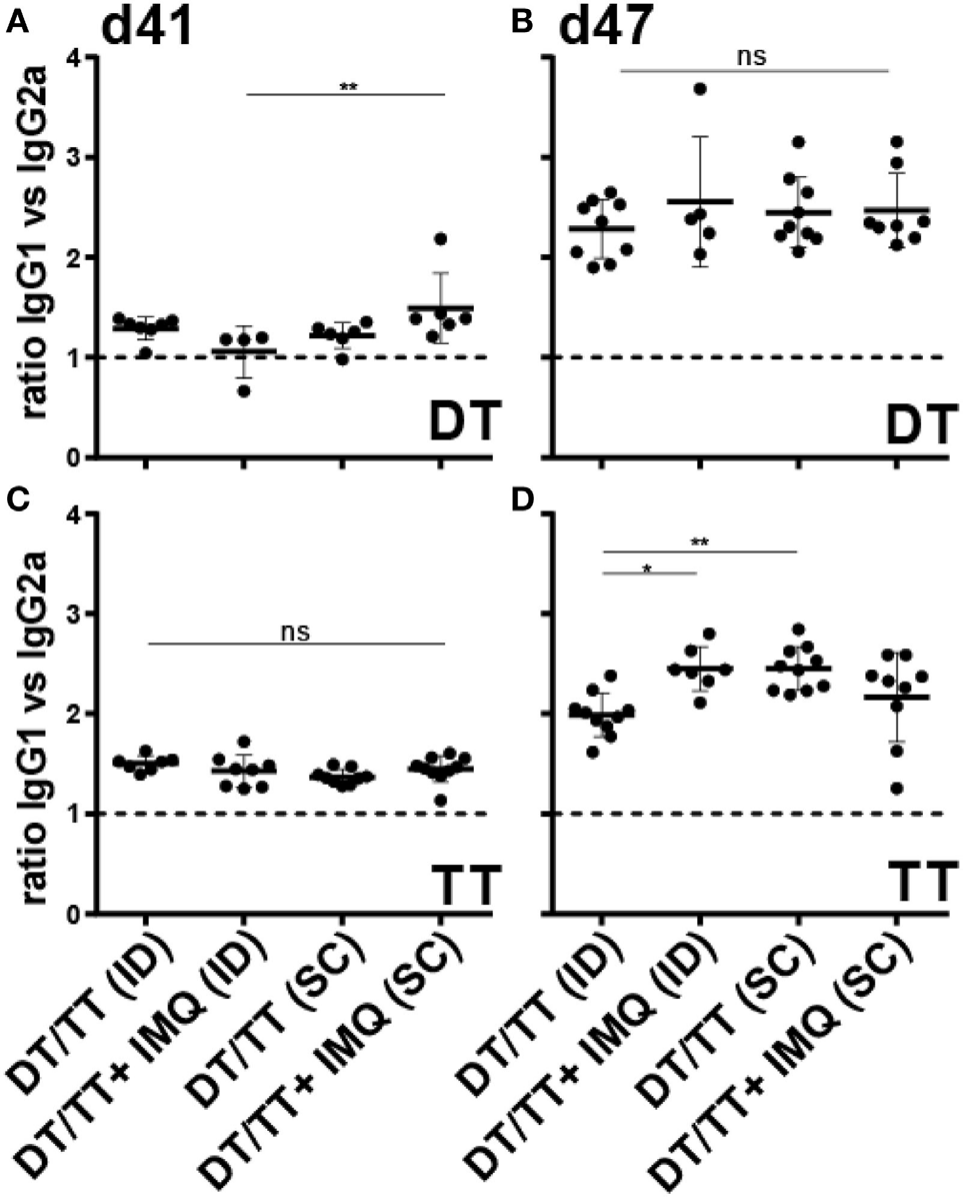
Ratio IgG1:IgG2a serum responses after microneedle-based intradermal (ID) and subcutaneous (SC) immunization with diphtheria toxoid (DT) **(A,B)** and tetanus toxoid (TT) **(C,D)** after the first boost **(A,C)** and second boost **(B,D)** vaccination. When antigens were adjuvanted with imiquimod (IMQ), only half of the antigen dose was applied compared with non-adjuvanted groups. Kruskal–Wallis test with Dunn’s *post hoc* test was performed to determine statistical differences between ratios.

To determine whether IMQ skews the induced DT- and TT-specific response, relative quantities of the IgG isotypes IgG2a and IgG1, which serve as markers for T helper 1 and T helper 2 type lymphocytes, respectively, were determined after boost immunization. Relative quantities could not be calculated after prime immunization because of non-responders. Overall, ratios between DT and TT-specific IgG1:IgG2a, after first and second boost immunization, indicated that IgG1, and thus Th2 cell responses, prevailed (Figure 6; Figures S1B,C and S2B,C in Supplementary Material). Remarkably, addition of IMQ enhanced vaccine-induced DT-specific IgG1 responses (day 41) in SC but not in ID immunized mice. After the second booster immunization (day 47) these differences disappeared, and similar ratios between DT-specific IgG1:IgG2a were observed in all four immunization groups (Figure 6; Figures S1B,C and S2B,C in Supplementary Material). For TT specific isotypes, the kinetics were different. While after the first boost immunization, in all mouse groups, ratios between IgG1 and IgG2a were similar, both ID immunized mice receiving the adjuvanted vaccine and SC immunized mice receiving the unadjuvanted vaccine showed increased IgG1:IgG2a ratios after boost (Figure 6; Figures S1E,F and S2E,F in Supplementary Material). Taken together, although no major differences in IgG isotype ratios between groups were observed, these data demonstrate that followed vaccination regimen induces a predominantly Th2-skewed lymphocyte response.

## Discussion

Nowadays, many microneedle technologies are under investigation for their potential future application in ID immunization, because they can be used to deliver drugs and vaccines in a minimally invasive and potentially pain-free manner into the skin. In the landscape of microneedle technologies, nanoporous microneedles are relatively new and pose an immunization method that enables the loading of drug formulations into the pores of the MNAs, which are released *via* diffusion upon piercing of the microneedles into the skin. In this first immunological study, we show that ceramic alumina npMNAs can be used for ID immunization with subunit vaccines, aimed to elicit humoral responses.

Nanoporous microneedle array strength is an important characteristic contributing to the efficiency of skin piercing but is closely related to the porosity of the material (15). High porosity can weaken the material resulting in breakage of the microneedle tips and thereby resulting in less efficient piercing of the skin. When designing npMNAs for the delivery of proteins or subunit vaccines, larger pores are necessary. The use of alumina (Al_2_O_3_) AKP30 particles for the production of npMNAs results in approximately 40% porosity, with an average pore size of 80 nm (13) and such npMNAs have sufficient strength to repeatedly penetrate the skin without breaking (10). Furthermore, it was previously shown that nanoporous microneedles can be loaded with small molecules and nanoparticles with sizes up to 100 nm (10). Alumina npMNAs, although with different microneedle geometry, but the same pore size distribution, have previously been loaded with small molecules (10) and short peptides (17). However, peptides are in general not ideal for prophylactic vaccination, because they contain only one minimal T or B cell epitope. Here, we show for the first time successful loading of npMNAs with the subunit vaccine proteins DT and TT, and the subsequent release of these antigens from npMNAs into skin, which potentially give rise to various epitopes.

Nanoporous microneedle arrays have an interconnected porous structured network throughout both the backplate reservoir and the microneedles (21), which allows for the loading of drug formulations in both the microneedles as well as the backplate reservoir. While loading relatively high amounts of drug formulations into the npMNAs (including backplate) can have advantages, for vaccines it might be disadvantageous. The diffusion of vaccines, or other biomacromolecules, from the backplate *via* the tips into the skin is a time consuming process and might therefore lead to a less efficient delivery efficiency of expensive molecules. In this study, we successfully developed a procedure to load only the microneedles tips of the npMNAs, post production of the npMNA, resulting in a relatively efficient use of vaccine formulations and allowing for limited application time on the skin. In addition, absorption of a formulation (by porous microneedles) could be favorable over adsorption onto (or coating of) the surface of, for instance, solid microneedles, because (1) the microneedle tip sharpness is retained and (2) potentially less excipients are required to retain the immunogenicity of a vaccine. Coating of solid MNAs generally requires a thick drug-containing layer to achieve the required amounts of drug/vaccine loading, and this could reduce the sharpness of the tips and thereby their skin piercing ability (22, 23). Furthermore, several excipients are required to adsorb the coating onto the microneedle surface and retain the immunogenicity of vaccines (24, 25). On the other hand, dissolving microneedles, wherein the drug/vaccine is embedded in the microneedle matrix require a more complex loading strategy in which the vaccine and excipients are added during the preparation phase. Hence, using dissolving microneedle technologies to only load the microneedle tips with a drug/vaccine is more challenging as compared with using porous microneedles. Therefore, npMNAs can be advantageous over coated and dissolving MNAs.

The release of DT, TT, and IMQ from drug-loaded npMNAs, was determined after incubating the drug-loaded npMNAs in release buffer *in vitro*. Indeed, around 30% of the vaccine antigens that were loaded into the npMNAs were released in buffer after 30 min. To establish if the vaccine subunit antigens were also released from the npMNAs after they penetrated the skin, and to quantify the amount of antigen delivered, *ex vivo* mouse ears were pierced with fluorescently labeled antigen-loaded npMNAs. The amount of delivered antigen was quantified, from which the release and delivery efficiencies were calculated. The ID delivery efficiency of DT was around 20%, and about 50% for TT. When the amounts of antigens that were released into the skin are correlated to the human vaccination dose (26, 27), the estimated diameter of the circular npMNA that needs to be used for human application should be 2.3 and 2.5 cm for DT and TT, respectively. This indicates a feasible size to deliver the corresponding vaccine doses.

Because our ceramic npMNAs have an average pore size of 80 nm, preferably low-molecular weight adjuvants are co-formulated into the nanopores of the npMNAs. For example, alum is a potent (micrometer-sized) adjuvant for the induction of humoral immune responses, but it cannot fit into the nanopores of our npMNAs. Besides, alum causes granuloma formation and should therefore not be used as an ID adjuvant (28).

In this study, IMQ, which is a toll-like receptor 7 agonist with a molecular weight of 240 g/mol, was chosen as an adjuvant. IMQ is extensively researched for its adjuvanticity (19, 29, 30), and as TLR agonist holds promise for vaccination approaches, because it induces the release of pro-inflammatory cytokines (31, 32). The cytokine profile induced by IMQ specifically favors Th1 over Th2 type responses (33, 34), and thereby the induction of a cellular immune response. Furthermore, it has been shown that topical application, rather than ID injection, activated antigen-presenting cells in skin explants (35). IMQ 5% cream (Aldara) already has FDA approval for topical use for the treatment of warts, actinic keratinosis and superficial basal cell carcinoma. Together, this makes IMQ a potent and attractive adjuvant for ID immunization. In the adjuvanted vaccine formulation, half of the antigen dose was used to investigate whether the vaccine dose could be decreased using npMNAs with an adjuvant that fits into the nanopores of the npMNAs. Since no enhanced immunogenicity was observed using IMQ, we cannot make any statement about the adjuvanticity of IMQ using our npMNAs in combination with DT and TT. Furthermore, we found that, in most immunization groups, IMQ did not have a significant effect on ratios of DT- and TT-specific IgG1:IgG2 responses, i.e., IgG1 responses prevailed in all groups. Only in mice receiving a triple immunization using npMNAs, enhanced TT-specific IgG1:IgG2a ratios were observed in the IMQ-adjuvanted vaccine groups. Taken together, these findings suggest that npMNA-mediated ID immunization with IMQ-adjuvanted vaccine predominantly induces Th2, and not Th1 responses. When further exploring the immunological potential of ceramic npMNAs and selecting future adjuvants, one should consider the limited pore size of the npMNAs. Therefore, in future studies aimed to optimize microneedle-based intradermally delivered vaccines, we will focus on use of low-molecular weight adjuvants, such as cGAMP (36), for co-formulation into the nanopores of npNMAs.

Finally, in this study, it was shown that strong antibody responses were induced without using an adjuvant. The antibody responses obtained in our study are in line with the ones described in literature (37, 38). For example, in previous studies doses of 0.3 µg [unadjuvanted DT (37)] and 0.1 µg [unadjuvanted DT and TT (38)] have been used for the induction of antibody responses (in mice and rats, respectively), which resulted in antibody-specific log titers of 4–5. In our study, similar antigen-specific antibody titers were obtained (log titers of approximately 4) against DT and TT using a similar dose. Therefore, this study shows the potential of npMNAs for microneedle-based ID vaccination using subunit vaccines.

## Conclusion

Taken together, in this study we show that ceramic nanoporous microneedles are strong enough to repeatedly penetrate the skin and that they can be loaded with protein subunit vaccines such as DT and TT. After skin piercing with antigen-loaded npMNAs, the antigens are intradermally delivered, which resulted in an induction of antigen-specific antibody responses. In conclusion, we show for the first time the potential of npMNAs for ID immunization with subunit vaccines, which opens possibilities for future ID vaccination designs.

## Ethics Statement

Ethical approval was obtained from Animal Ethics Committee from Utrecht University, the Netherlands (DEC #2014.II.11.080).

## Author Contributions

Substantial contributions to the conception was done by KM, PV, and AS and design of the work by KM, AS, and AP. Acquisition, analysis, and interpretation of data by AP, KM, AG, NK, and PK. Drafting the manuscript by AG and KM. All the authors revised the work and gave final approval of the version to be published.

## Conflict of Interest Statement

KM is co-owner of uPRAX Microsolutions. The other authors declare that they have no commercial or financial relationships that could be construed as a potential conflict of interest.

## Abbreviations

IMQ, imiquimod; MNA, microneedle array; npMNA, nanoporous microneedle array; IgG, immune globulin G; DT, diphtheria toxoid; TT, tetanus toxoid; TLR, toll-like receptor; DLS, dynamic light scatter; Lf, limits of flocculation; HPLC, high-performance liquid chromatography; TFA, trifluoroacetic acid; TMB, 3,3′,5,5′-tetramethylbenzidine; BSA, bovine serum albumin; PBS, phosphate-buffered saline; ACN, acetonitrile; HRP, horseradish peroxidase; OD, optical density; PBST, 0.01% Tween 20 in PBS.
