# Supplementary material for: Nanoporous Microneedle Arrays Effectively Induce Antibody Responses against Diphtheria and Tetanus Toxoid
Source: Front Immunol. 2017 Dec 13;8:1789. doi: 10.3389/fimmu.2017.01789 (PMC5770646; doi:10.3389/fimmu.2017.01789)
Supplement: Supplementary file 1 [file Data_Sheet_1.PDF]

Nanoporous Microneedle Arrays to Efficiently Induce Antibody Responses against Diphtheria and Tetanus Toxoid

SUPPLEMENTARY INFORMATION

Anne Marit de Groot[1Ŧ], Anouk C.M. Platteel[1Ŧ], Nico Kuijt[2], Peter J.S. van Kooten[1], Pieter Jan Vos[2], Alice J.A.M. Sijts[1], Koen van der Maaden[2*]*

[1] Department of Infectious Diseases and Immunology, Faculty of Veterinary Sciences, Utrecht University, the Netherlands
[2] MyLife Technologies B.V., Leiden, the Netherlands

[Ŧ]Equal contribution
*Corresponding authors

Correspondence should be addressed to e.j.a.m.sijts@uu.nl and maaden@mylifetechnologies.nl

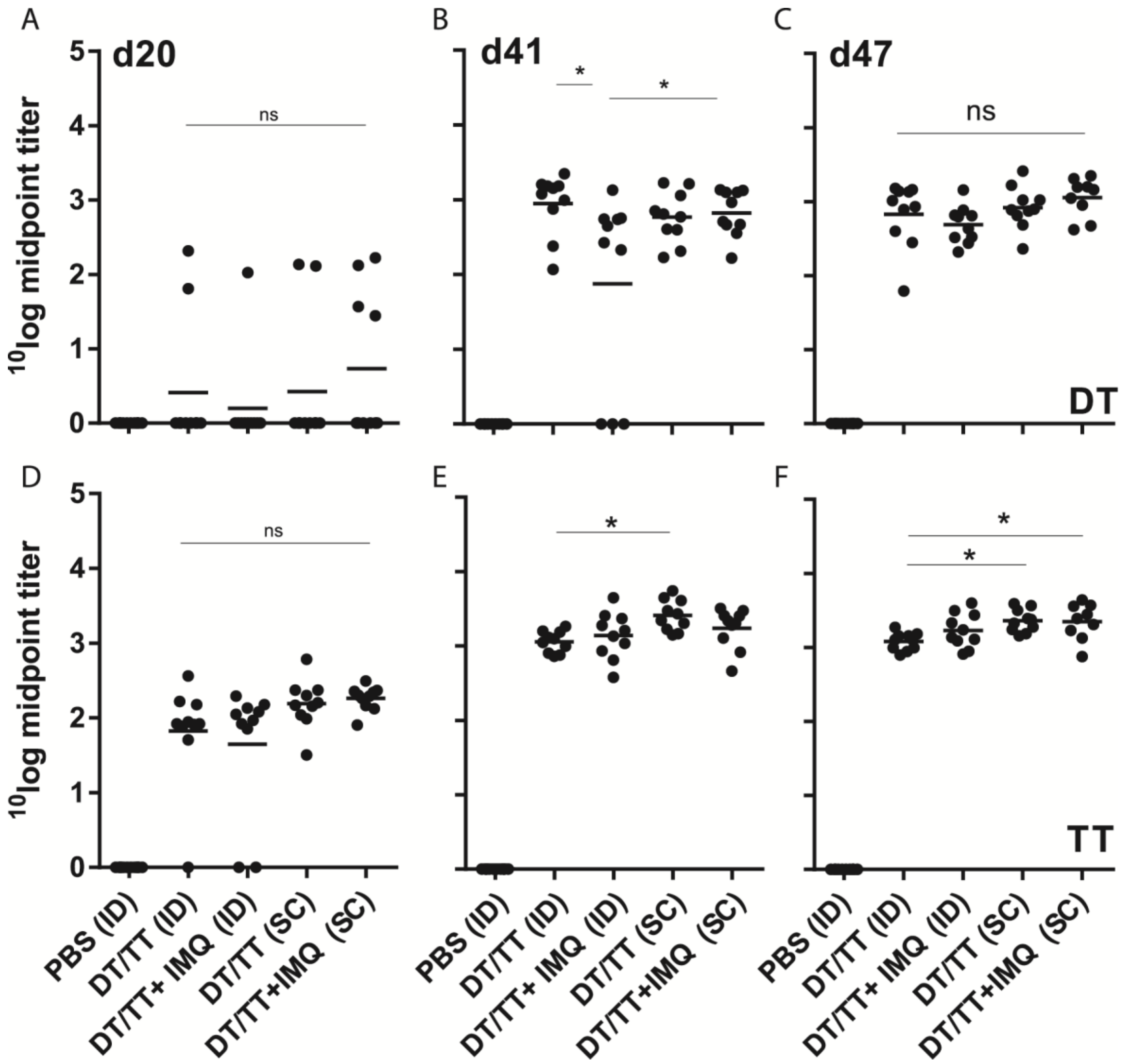

Supplementary Figure 1: Serum IgG1 responses (mean + individual results) after ID or SC immunization with PBS or DT and TT, both routes with or without Imiquimod. When imiquimod was added only half doses of antigens was used. IgG1 responses were detected against DT antigens after prima (A), 1st boost (B) or after 2nd boost (C) and TT antigens (D-F). Kruskall-Wallis test with Dunn's post-hoc test were performed to deter

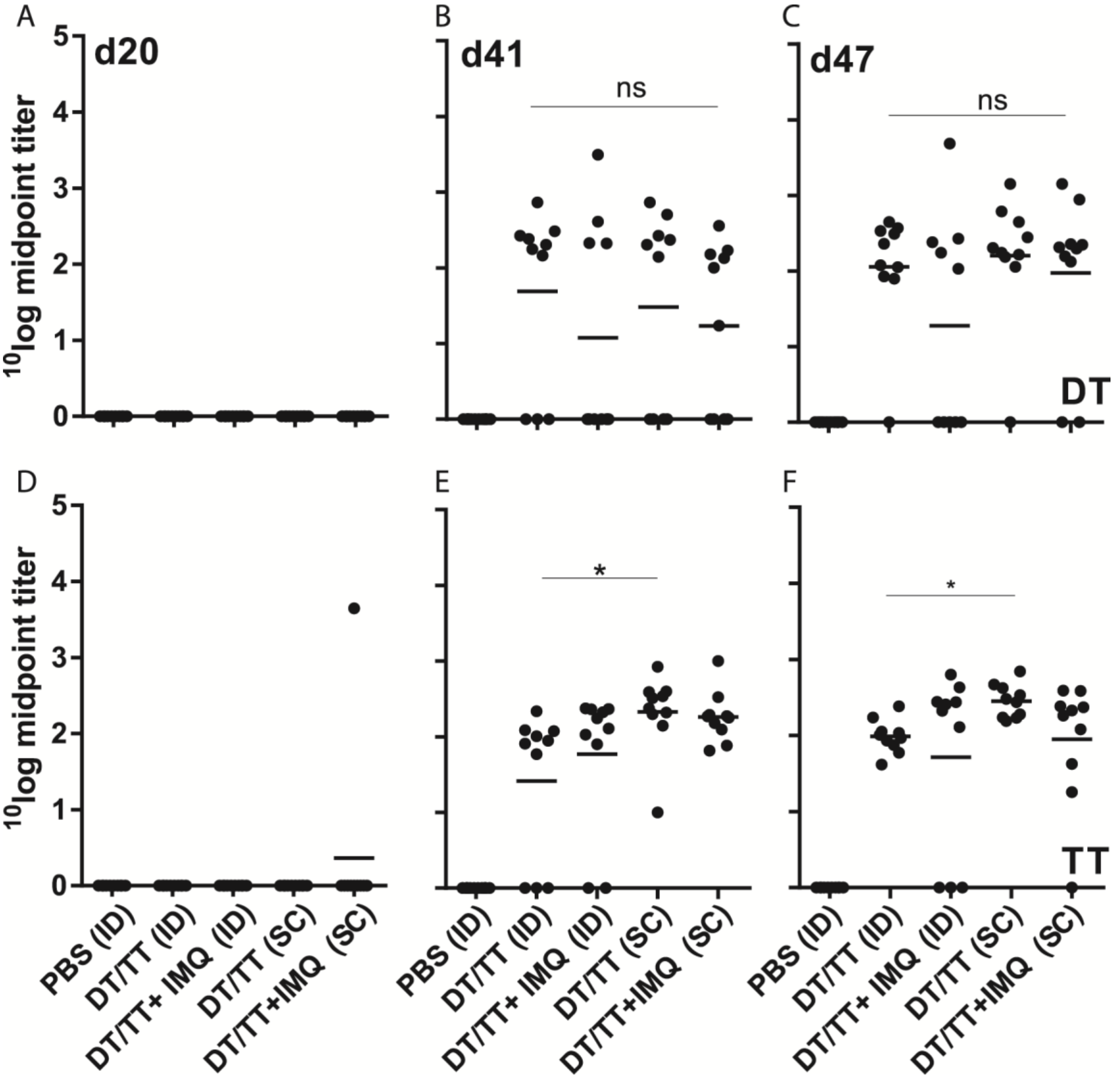

Supplementary Figure 2: Serum IgG2a responses (mean + individual results) after ID or SC immunization with PBS or DT and TT, both routes with or without Imiquimod. When imiquimod was added only half doses of antigens was used. IgG2a responses were detected against DT antigens after prima (A), 1st boost (B) or after 2nd boost (C) and TT antigens (D-F). Kruskall-Wallis test with Dunn's post-hoc test were performed to determine statistical differences of midpoint titers determined using 4 different titers dilutions.
